# Telehealth: Increasing Access to High Quality Care by Expanding the Role of Technology in Correctional Medicine

**DOI:** 10.3390/jcm6020020

**Published:** 2017-02-13

**Authors:** Jeremy D. Young, Melissa E. Badowski

**Affiliations:** 1 Division of Infectious Diseases, Department of Medicine, University of Illinois at Chicago, Immunology and International Medicine, University of Illinois at Chicago, 808 S. Wood Street, #888, Chicago, IL 60612, USA; 2 Department of Pharmacy Practice, College of Pharmacy, University of Illinois at Chicago; Chicago, IL 60612, USA; badowski@uic.edu

**Keywords:** telehealth, telemedicine, correctional healthcare

## Abstract

The United States (US) has a large correctional population. However, many incarcerated persons lack access to evidence-based, up-to-date medical care, particularly by subspecialty providers, due to limitations of geography, travel, cost and other resources. The use of telehealth technologies can remove these barriers, increasing access to high quality, multidisciplinary care. Studies have shown that, with telemedicine, timely triage and medical management can be provided across many disciplines, which may lead to improved clinical outcomes and significant cost savings.

## 1. Disease and Healthcare in Correctional Facilities

The United States (US) incarcerates a relatively high proportion of its convicted criminal offenders. At the end of 2014, there were 1,561,500 prisoners housed in state and federal correctional facilities, amounting to nearly 500 inmates per 100,000 US residents [1]. The total combined federal, state and local correctional population in 2014—including those on probation and parole—was 6,851,000 persons, consistently around seven million for more than a decade [2]. The US imprisons more individuals per population than any other nation in the world, with a 239% increase during the 1990s, stimulated in large part by the institution of harsher sentencing for non-violent drug offenses [3]. This large correctional population contains a diverse array of individuals, many of whom suffer from both acute and chronic diseases, who must be diagnosed and managed while incarcerated in jails or prisons. However, correctional settings often lack the appropriate resources to provide timely, expert medical care. Some correctional facilities transport inmates to local hospitals or clinics for medical care; however, this is costly, consumes personnel resources, presents a risk of flight, and is not always feasible.

Access to subspecialty care, in particular, is often lacking in facilities across the country. By design, prison facilities are frequently located in rural areas, relatively far from larger cities with tertiary care providers and consultants. Incarcerated individuals often do not have easy, or any, access to medical professionals with subspecialty training and experience due to the common barriers of geography, limited transportation and cost. If experts are involved directly with patient care and provide high quality, up-to-date, evidence-based medical care—particularly in experienced, multidisciplinary care teams—there may be an improvement in disease-related morbidity and survival for persons with a variety of chronic diseases. The literature contains myriad examples of complex, chronic disease states for which subspecialist care positively effects patient outcomes, including human immunodeficiency virus (HIV) [4,5,6,7,8], diabetes mellitus [9,10,11], inflammatory bowel disease [12], cystic fibrosis [13,14], rheumatoid arthritis [15,16,17] and many others.

Unlike the developing world, where clinician shortages and a lack of diagnostic and therapeutic modalities may exist, the US contains an abundance of healthcare resources and clinician expertise. However, feasibly connecting to care remains an issue for many of the underserved. For the incarcerated, technology-based solutions must be utilized to improve access to care, connecting patients with providers in a way that removes geographic barriers and the healthcare restrictions of the correctional environment. Any reasonable solution must provide quality care, fit within often restrictive federal, state and county budgets, and traverse the often large geographic areas separating healthcare providers from correctional facilities. Evidence shows that up to 50% of ambulatory care visits could safely and reliably be accomplished via telemedicine [18], suggesting that the use of telehealth technologies could have a major impact on the future of healthcare, particularly for traditionally underserved individuals and populations.

Clinicians can provide outstanding, comprehensive—and much-needed—care by harnessing telehealth technologies in the care of inmates. The incarcerated population has a significantly elevated prevalence of many chronic medical conditions as compared with the general population, including HIV and hepatitis C, both associated with overlapping high-risk behaviors for incarceration, such as substance abuse and commercial sex work. An estimated 20%–30% of those in US jails and prisons are infected with hepatitis C [19,20], with an HIV prevalence approximately three times greater than the general population [21,22]. High rates of other serious medical conditions, such as chronic obstructive pulmonary disease (COPD), asthma and hypertension [23], also exist in the correctional setting. However, these large populations are often managed by a single on-site physician and/or physician extender. Inmates are disproportionately affected with psychiatric and substance abuse issues as well, with an estimated 40%–60% of offenders having some mental health disorder [24,25], but the needs frequently outweigh the availability of mental health professionals. Indeed, there is currently a crisis-level shortage of quality mental healthcare in US correctional facilities, with a widespread call for an increase in resources [26]. When acute medical complaints arise, the use of teletriage may lead to more appropriate decision-making regarding the transportation of ill inmates to Emergency Departments, potentially decreasing the unnecessary use of resources while also evaluating those in need of immediate evaluation or inpatient admission with expediency.

## 2. Methods

Literature searches of the Pubmed/Medline (through 12 January 2017) and Google Scholar™ databases were performed using the search terms telehealth, telemedicine, inmate, prison, prisoner, and corrections. Only English-language articles, published in indexed journals, pertaining to the search terms were evaluated.

## 3. Uses and Benefits of Telehealth in Correctional Care

The terms telehealth and telemedicine are often used synonymously but there is a slight distinction. Telehealth is a general term that encompasses telemedicine and uses telecommunications to provide healthcare, patient and professional education, and public health data [27,28]. The goal of telemedicine is to improve patient health outcomes through the use of two-way interaction between the patient—located at the originating site—and a clinician at a distant site.

Generally, telemedicine modalities fall into one of two categories: synchronous or asynchronous. In synchronous telemedicine, a confidential, interactive, two-way audio and video connection replaces the in-person, face-to-face visit, using specialized equipment to perform an accurate and reliable history and physical exam. Synchronous telemedicine models are typically used to manage acute and chronic diseases that rely significantly on a real-time patient interaction or the physical exam, such as the management of chronic infectious diseases, pulmonary medicine, diabetes management and telepsychiatry.

Asynchronous telemedicine models—also known as “store and forward”—transmit medical data, including documented histories, medical records, laboratory results, and high-definition photos of physical exam findings and radiographic data for a medical provider to review. This model is best used when experts are assessing a very specific physical exam finding or radiographic study and the plan or recommendations are not urgent. Teledermatology and teleradiology often employ asynchronous technologies in their practices, electronically storing images, such as photos of rashes or magnetic resonance imaging (MRI), and forwarding them to physicians for assistance with diagnosis and advice regarding further diagnostic studies and therapeutic options. There are myriad ways in which technology can be used to manage patients successfully, and each program should be designed for the specific needs of any given clinic. Certainly, both synchronous and asynchronous methods can be used in the correctional setting, each when appropriate.

Encryption software, high-definition cameras, monitors, and equipment used to perform physical exams, such as electronic stethoscopes, otoscopes and ophthalmoscopes, are widely available and generally affordable. In practice, synchronous telemedicine has very few clinical limitations, and several studies in the prison population have reported successful outcomes when using telemedicine for psychiatric management [29,30], surgical services [31] and emergency medicine [32], with enhanced, timelier access to care, cost savings and high patient satisfaction [33]. In addition, the use of telemedicine for subspecialty, multidisciplinary care of prisoners with HIV has been shown to result in a greater virologic suppression and rise in CD4+ T-lymphocyte counts [34], and improved laboratory markers associated with morbidity, mortality and transmission [35,36,37,38], and to enhance the coordination of care between services for people living with HIV (PLWH) [39]. A research group in New Mexico assessed sustained virologic response in the treatment of hepatitis C infection, and found no difference in outcomes between an in-person university clinic and management with telemedicine [40], providing further evidence for the efficacy of telehealth.

In one study of a prison telemedicine intervention, two adolescent correctional facilities experienced a 57% decrease in overall wait time for a medical care referral, and a significantly decreased time from referral to treatment after the program was implemented. In the same study, outpatient visits increased 40% via telemedicine, and emergency room visits significantly decreased by year two [41]. These data suggest that a telehealth program could increase access to care, lower unnecessary costs, allow inmates to be seen sooner, and facilitate the initiation of appropriate management, potentially improving morbidity, and even mortality.

Furthermore, additional benefits to utilizing telemedicine in the correctional setting include eliminating the need to transport each inmate to their medical appointment. This would remove the requirement for multiple guards and special transport equipment for moving prisoners to conventional healthcare settings for specialty care. In addition, this would reduce poor public acceptance of shackled prisoners in the waiting area with non-prisoner patients.

## 4. Implementing an Effective and Sustainable Telehealth Program

Although the American Telemedicine Association (ATA) developed a set of core operational standards for telehealth services involving provider and patient interactions as well as a guideline for primary and urgent care [42,43], designing, implementing and maintaining a telehealth program can be rife with complexity. However, if experts are involved in each aspect, with clear protocols, open communication between team members, and frequent program evaluation and quality assurance, an outstanding telehealth program can be implemented and maintained. The key is to recognize the importance of every component vital to a successful telemedicine clinic: purchasing and installing the correct equipment and technology, ensuring reliable connectivity, furnishing adequate clinic space at the originating and distant sites, providing quality clinical care, acknowledging the limitations of telehealth, assuring patient confidentiality consistent with the Health Insurance Portability and Accountability Act (HIPAA) of 1996, maintaining patient satisfaction, appropriately creating and storing medical records, communicating well with other healthcare providers at originating and distant sites, designing a sustainable fiscal model for telehealth clinics, remaining cognizant of individual state and insurance regulations and restrictions on the use of telehealth and, for some programs, instituting teaching and research programs. For some, a multidisciplinary approach to care is also an important consideration. For example, the HIV Prison Telemedicine Clinic at the University of Illinois Hospital and Health Sciences System integrates an infectious diseases physician, an HIV-trained clinical pharmacist and a case manager in a real-time, synchronous patient interaction to address all aspects of optimal HIV care. Patients are screened on intake for the presence or history of HIV/AIDS. The inmate is then referred to the telemedicine clinic within 30 days from intake. For new patients, consents are reviewed and signed prior to receiving treatment in the telemedicine clinic. Patients are evaluated on a routine basis to evaluate the safety and efficacy of their antiretroviral therapy, consistent with the national guidelines. A nurse at the far site assists in the clinical encounter and physical exam for placement of the telephonic stethoscope or high-definition exam camera. Financial support for programs involving telemedicine or mobile health (mHealth) is a vital consideration, and can be a particularly complex undertaking when instituting a correctional program. Federal, state and county budgets rarely make room for altruistic pursuits. Therefore, the importance of designing a sustainable business model for the provision of telehealth services cannot be understated. Some of the costs to consider include the time of physicians, physician extenders and nurses, clinic space, drug costs, and the expense of purchasing and maintaining equipment, encryption software and networks. Some programs fund their operations via billing. States can be billed for telemedicine clinic visits, but this must be arranged in a contractual manner, and many state budgets do not allow for the additional expense of telehealth. An increasing number of states are passing legislation to expand reimbursement to include telemedicine, with 29 states and the District of Columbia currently mandating parity in billing for private insurance to reimburse for telemedicine visits—and an additional eight states with proposed parity bills under current consideration—and 47 state Medicaid programs providing some coverage for telemedicine [44]. However, these funding sources have highly variable regulations and would only apply to the non-incarcerated population. The expense of correctional healthcare can be partially offset through telehealth-related cost avoidance including lower drug costs, removing the need for expensive transportation to in-person clinics, and improved medical care, ultimately resulting in fewer complications, hospitalizations and lawsuits. Analyses of true cost savings can be difficult, but are an important aspect of justifying many correctional programs.

While telehealth makes logical sense, with efficacy data in the literature, a great need for pursuing research in this new and expanding field persists. There are many issues to be explored including clinical outcomes, patient satisfaction, technology, clinic flow, limitations in diagnosis and management, training, legislation, reimbursement and cost savings. However, despite the growing body of literature in support the use of telemedicine in the correctional setting, and the call for increasing the use of telehealth technologies to improve access to care for inmates [45], too few centers are implementing technology-based medical care.

## 5. The Future of Telehealth in Corrections

The American Medical Association (AMA) has clearly acknowledged that providing telehealth services is really just healthcare, using a different, technology-based format. At its annual meeting in Chicago, AMA’s Board of Delegates agreed upon a public position statement on Ethical Practice in Telemedicine [46]. The policy affirms that physicians providing care via technology have the same ethical responsibilities as those providing care in brick and mortar environments. These responsibilities include providing competent care, respecting patient privacy and confidentiality, taking appropriate steps to ensure continuity of care, and following best practice guidelines.

An increased involvement of academia in the medical care of incarcerated individuals and populations has been called for in the literature [47,48]. In addition, observational studies, and even clinical trials, are vital emerging aspects of telehealth. Academic centers can also use telemedicine technologies for teaching a variety of healthcare providers, training students, residents and fellows, conducting multicenter conferences, and collaborating on research. Indeed, telehealth will play a major role in the future of healthcare, particularly for access-poor populations. If programs are developed with clear goals, clinical protocols and business models, with a clear understanding of the potential limitations of using telehealth technologies, outstanding care can be provided to the large and diverse correctional population. A need certainly exists.
